# Fungal Community Complexity and Stability in Clay Loam and Sandy Soils in Mangrove Ecosystems

**DOI:** 10.3390/jof11040262

**Published:** 2025-03-28

**Authors:** Shengyao Zhou, Xiaojie Deng, Rajapakshalage Thashikala Nethmini, Huaxian Zhao, Qing He, Gonglingxia Jiang, Qinghua Hou, Qingxiang Chen, Xiaolei Li, Ke Dong, Nan Li

**Affiliations:** 1 Tropical Ocean Environment in Western Coastal Waters Observation and Research Station of Guangdong Province, Key Laboratory of Climate, Resources and Environment in Continental Shelf Sea and Deep Sea of Department of Education of Guangdong Province, Department of Oceanography, Key Laboratory for Coastal Ocean Variation and Disaster Prediction, College of Ocean and Meteorology, Guangdong Ocean University, Zhanjiang 524088, China; syzhou001009@163.com (S.Z.); 15270735195@163.com (X.D.); nethmini1207@gmail.com (R.T.N.); 13531051195@163.com (Q.H.); stonecold_nan@163.com (G.J.); huaqingh@163.com (Q.H.); chenqxocean@163.com (Q.C.); xli@gdou.edu.cn (X.L.); 2 Key Laboratory of Ministry of Education for Environment Change and Resources Use in Beibu Gulf, Guangxi Key Laboratory of Earth Surface Processes and Intelligent Simulation, Nanning Normal University, Nanning 530001, China; zhx343@gmail.com; 3 Department of Biological Sciences, Kyonggi University, 154-42, Gwanggyosan-ro, Yeongtong-gu, Suwon-si 16227, Gyeonggi-do, Republic of Korea; dongke-007@163.com

**Keywords:** fungi, mangrove ecosystems, co-occurrence network, complexity, stability, clay loam, sandy soil

## Abstract

Soil fungi in mangroves are diverse and crucial for organic matter decomposition and element cycling. However, the drivers influencing network complexity and the stability of fungal communities across different mangrove soil habitats remain unclear. This study investigated the main factors driving the composition, diversity, complexity, and stability of fungal communities in clay loam and sandy soils in mangrove ecosystems. Results showed that *Dothideomycetes* and *Sordariomycetes* dominated in clay loam and sandy soils, respectively. Sandy soils exhibited higher alpha diversity than clay loam. Beta diversity analysis revealed significant differences in the fungal community structure between the two soil types. Network analysis demonstrated higher complexity and stability of fungal communities in clay loam than in sandy soil. Spearman’s correlation analysis revealed that NH_4_^+^-N and total nitrogen were the main factors affecting complexity and stability in clay loam, respectively. Partial least squares path modeling demonstrated that alpha diversity and soil properties were closely linked to the complexity and stability of fungal communities in clay loam, whereas beta diversity was the primary driver in sandy soil. Our study enhances the understanding of the mechanisms that maintain fungal diversity and community stability in mangrove ecosystems, with important implications for restoring vegetation in degraded areas.

## 1. Introduction

The fungal community in soil is rich and diverse, playing significant ecological roles as a key component of microbial communities, particularly in promoting plant nutrition, carbon cycling, and pathology [1,2]. In ecology, ecosystem complexity typically refers to the abundance of species and their interrelationships within the system, whereas stability refers to the ability of a system to maintain its function and structure despite external disturbances [3]. Human disturbances and ecological drivers inevitably impact biodiversity and fungal communities in various soil ecosystems. For example, S Luo et al. [4] found that increased grassland degradation caused by human disturbance on the Qinghai–Tibet Plateau led to a less complex and stable soil fungal community structure, highlighting the need to restore and protect the degradation-induced simplification of fungal communities. P Zhao et al. [5] identified soil nutrients as the determining factors of fungal network complexity and stability in Mongolian pine plantations, followed by climate conditions, which is helpful for understanding the interactions between plants and soil fungi. However, factors influencing fungal complexity and stability in saline soils remain poorly understood.

Mangrove forests hold significant ecological and economic values owing to their unique ecological environment. Mangroves provide ideal habitats for birds, insects, fish, and mammals [6] and protect coastlines by serving as natural barriers against tropical storms and waves [7]. The community structure and diversity of mangrove soil fungi are influenced by pH, salinity, and organic matter content [8,9,10]. In many cases, nutrient levels in mangrove soils are key ecological drivers of the fungal community structure [11,12]. For example, P Wei et al. [13] showed that phosphorus is important for fungal communities in mangrove ecosystems. Additionally, environments with abundant organic matter and appropriate pH levels promote greater fungal diversity in mangrove sediments [14].

Recent studies have highlighted the significant role of soil types in shaping fungal communities and their composition across various ecosystems. Different soil types create distinct ecological environments, which affect fungal diversity. For example, in forest soils, pH and carbon concentrations have strong indirect effects on fungal community composition [15]. Moreover, soil pH and available nutrients (such as nitrogen and phosphorus) are major drivers of fungal community shifts and diversity in desert grasslands [16]. However, fungal community composition and the key environmental factors driving its complexity and stability in various mangrove soil habitats remain poorly understood.

This study aimed to clarify the impact of different soil types on fungal community structures in mangrove soils in different habitats. Therefore, we collected clay loam and sandy soil samples from mangroves in Zhangjiang, Beihai, and Fangchenggang in southern China. The main objectives of this study were to (a) identify the species composition and diversity of fungal communities in clay loam and sandy mangrove soils, (b) evaluate the complexity and stability of fungal communities in these soils, and (c) clarify the key drivers determining the complexity and stability of fungal communities in clay loam and sandy mangrove soils. Overall, this study enhances our understanding of how different soil types influence fungal diversity maintenance mechanisms in mangrove ecosystems and provides valuable insights for the restoration and conservation of mangrove forests.

## 2. Materials and Methods

### 2.1. Study Area and Field Sampling

**Figure 1:**
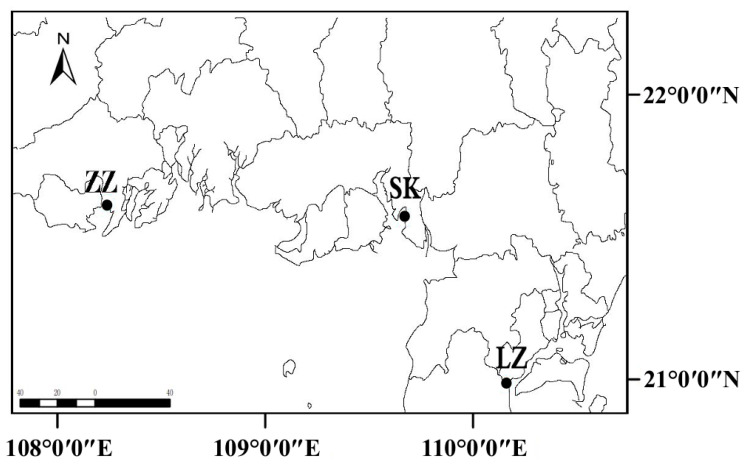
Distribution of the three mangrove sampling sites in southern China. ZZ: Zhenzhuwan mangrove site, sandy soil; SK: Shankou mangrove site, sandy soil; LZ: Leizhou mangrove site, clay loam. Two sites, SK and ZZ, are located in Guangxi province, while LZ is located in Guangdong province.

The sampling sites were located in coastal mangrove nature reserves in Zhenzhu Bay (ZZ), Shankou (SK), and Leizhou (LZ) in southern China (Figure 1). In July 2019, we collected 75 individual samples from the three accessible mangrove sites. Five plots (5 × 5 m^2^) were established in each region for sampling. Sediment samples were collected from the surface layer (1–2 cm) and divided into three categories based on sand content: 0–10% (muddy or clay soil), 10–60% (mud–sand mixed soil or clay loam), and 60–100% (sandy soil) [17]. The LZ sample consisted of clay loam. Samples SK and ZZ were categorized as sandy soil. The salinity of the LZ sample is higher than that of the SK and ZZ samples. The content of clay and silt in the LZ sample is significantly higher than that in the SK and ZZ samples. The content of sand in the LZ sample is lower than that in the SK and ZZ samples.

All individual collections were stored at −80 °C in case of DNA extraction for sequencing analysis.

### 2.2. Biochemical Factor Analyses

Biochemical parameters such as temperature, salinity, pH, and dissolved oxygen (DO) were measured in each region using a portable meter (556 MPS, YSI, Yellow Springs, OH, USA) for mangrove sediment samples. A Malvern Mastersizer 2000 (Malvern, UK) was used to determine the soil texture (sand, silt, and clay). Measurements were taken for total organic carbon (TOC), total carbon (TC), total sulfur (TS), total nitrogen (TN), total phosphorus (TP), total inorganic carbon (TIC), PO_4_^3−^, SO_4_^2−^, and inorganic nitrogen (NO_2_^−^-N, NO_3_^−^-N, and NH_4_^+^-N) in the laboratory.

### 2.3. DNA Extraction and PCR Amplification

The DNeasy PowerOil Pro kit (QIAGEN, Hilden, Germany) was used to extract DNA from 75 frozen mangrove sediment samples (0.25 g), following the manufacturer’s protocols of the reagent kit. DNA integrity and purity were evaluated using a Nanodrop-2000 spectrophotometer (Thermo Fisher Scientific, Waltham, MA, USA). The DNA samples were stored at −80 °C. For fungal DNA amplification of the ITS1 region, primers ITS1F (5′-ACTTGGTCATTTAGAGGAAGTAA-3′) and ITS2 (5′-BGCTGCGTTCTT-CATCGATGC-3′) were used. The amplification process was carried out with the following cycling conditions: 95 °C for 5 min, followed by 30 cycles of 94 °C for 30 s, 50 °C for 45 s, 72 °C for 2 min, and a final extension at 72 °C for 10 min. The quality of PCR products was tested using 2% agarose gel electrophoresis and a Nanodrop 2000 spectrophotometer (Thermo Scientific, Wilmington, DE, USA). Sequencing samples were prepared using a TruSeq DNA kit (Illumina, San Diego, CA, USA) following the manufacturer’s guidelines and sequenced using the Illumina MiSeq platform (San Diego, CA, USA) with a 2 × 250 bp Reagent Kit v2.

### 2.4. Bioinformatics Analysis

Primer mismatches or lengths of <275 bp, low-quality reads (quality scores < 30), and barcode sequences were removed using the DADA2 denoising method in QIIME 2. Amplicon sequence variants generated from the Illumina Scale amplicon data were used for further analysis. Taxonomic classification was conducted using the Ribosomal Database Project (RDP) classifier with a confidence threshold set at 80%. To reduce bias, the original sequence data were deposited in GenBank under BioProject Accession PRJNA771484.

### 2.5. Statistical Analyses

Statistical analyses were performed using the R software (version 4.3.2) (http://www.r-project.org/ accessed on 18 January 2019). The indices of Shannon was calculated using the “vegan” package (version 2.6-10). The Shannon index was used to represent alpha diversity. Principal coordinate analysis (PcoA) and analysis of similarity (ANOSIM) were performed to evaluate the differences in community structures among different samples based on the Bray–Curtis distance using the “vegan” package and visualized using the “ggplot2” package (version 3.5.1). Network analysis was performed using the “Hmisc” package (version 5.2-3) and visualized using the Gephi 0.9.2 software. Average degree measured the average interaction strength of microorganisms in the network. Density was calculated based on the tightness and complexity of the network. Modularity quantified the degree to which a network is compartmentalized into different modules. To determine the complexity and stability of the samples, total cohesion was calculated to reflect complexity. Positive cohesion, resulting from positive pairwise correlations, may represent the extent of cooperative behaviors within a sample, while negative cohesion could suggest the level of competitive interactions among community members [18]. Robustness was considered as an indicator of stability. Network robustness was quantified as the proportion of the remaining species in this network after random or targeted node removal. We measured the robustness when 50% of random nodes or five module hubs were removed [18]. As described in [18,19], $\mathrm{Average Degree}=\frac{1}{n}\sum_{i=1}^{n}k_{i}$, where *k_i_* is the degree of node *i* and *n* is the number of nodes; $\mathrm{Density}=\frac{l}{l_{exp}}=\frac{2l}{n(n-1)}$, where *l* is the sum of total links and *l_exp_* is the number of possible links; $\mathrm{Cohesion}=\sum_{i=1}^{m}{abundance}_{i}\times {connectedness}_{i}$, where *m* is the total number of taxa in a community; $\mathrm{Robustness}=\frac{\sum_{j\neq i}b_{j}s_{ij}}{\sum_{j\neq i}b_{j}}$, where *b_j_* is the relative abundance of species *j* and *s_ij_* is the association strength between species *i* and *j*, which is calculated using Pearson’s correlation coefficient. Geographical distance matrices were computed using the distGeo function from the “geosphere” package (version 1.5-20) in R, based on the WGS84 ellipsoid model. Correlations were calculated using Spearman’s rank method. Mantel test analysis was conducted to identify environmental factors that affect the structure of sediment fungal communities using the “vegan” package. Spearman heatmaps were generated using the “pheatmap” package (version 1.0.12) to display the relationship among environmental factors, complexity, and stability. Linear regression analysis was performed using the “ggplot2” (version 3.5.1) and “ggpmisc” packages (version 0.6.1). Partial least squares path modeling (PLS-PM) was conducted to determine the direct and indirect contributions of driving factors on complexity and stability using the “plspm” package (version 0.5.1).

## 3. Results

### 3.1. Composition and Diversity of Fungal Communities in Mangrove Clay Loam and Sandy Soil

**Figure 2:**
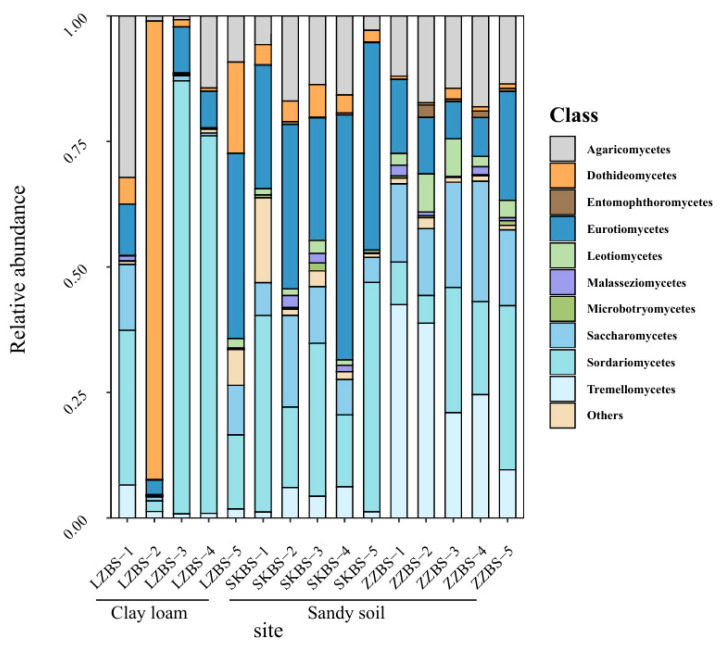
Relative abundance of fungi in mangrove clay loam and sandy soil (class level). BS: bulk sample; 1–5: site name.

In this study, 75 mangrove sediment samples were collected from the clay loam and sandy soil sites. Ultimately, 399 and 150 high-quality sequences were obtained. In the clay loam samples, *Dothideomycetes* (64%) was the most dominant class, followed by *Sordariomycetes* (19%) and *Eurotiomycetes* (8%). In contrast, in the sandy soil samples, the abundances of *Sordariomycetes* (25%) and *Eurotiomycetes* (23%) ranked first and second, respectively. The sandy soil samples were obtained from the ZZ and SK sites. At the ZZ site, *Tremellomycetes* was the dominant class (25%), followed by *Sordariomycetes* (20%) and *Saccharomycetes* (17%). At the SK site, the community was mainly composed of *Eurotiomycetes* (32%), followed by *Sordariomycetes* (30%) and *Agaricomycetes* (11%) (Figure 2). These results indicate that environmental heterogeneity influences the composition of the mangrove fungal community.

**Figure 3:**
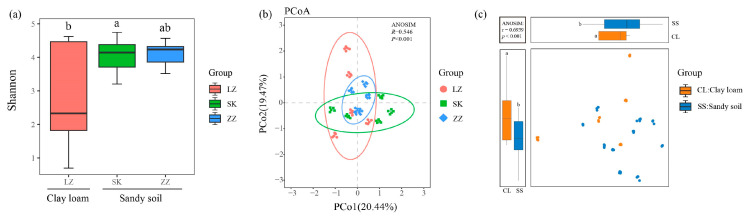
(**a**) Alpha diversity (Shannon index) presented by boxplots. In the boxplots, the upper whisker represents the maximum value; the upper line of the box represents upper quartile; the center line inside the box represents the median; the lower line of the box represents the lower quartile, and the lower whisker represents the minimum value. Different lowercase letters represent significant differences (*p* < 0.05), while the same lowercase letters indicate no significant differences (*p* > 0.05). (**b**) Differences in fungal community structures across different sites (PCoA). (**c**) Differences in fungal community structures between clay loam and sandy soils. “a” and “b” are commonly used to denote statistically significant differences between groups.

Alpha diversity analysis demonstrated that the Shannon index of the sandy soil sample was higher than that of the clay loam. Additionally, fungi from the sandy soil at site ZZ exhibited significantly greater alpha diversity than those in the samples from other sites. The Shannon index of the sandy soil ZZ site was the highest, whereas that of the clay loam LZ site was the lowest. Intergroup differences were observed between the clay loam LZ and SK sites, but no intergroup differences were observed when compared to the sandy soil ZZ site (Figure 3a). PCoA of beta diversity (Bray–Curtis distance) revealed differences in fungal community structures between mangrove soils at different locations, clay loam, and sandy soils (Figure 3b,c). The first ordination axis (PCo1) and second ordination axis (PCo2) explained 20.44% and 19.47% of the community variation, respectively. The ANOSIM test confirmed significant differences in the fungal community structure among mangrove sediments from different sites (R = 0.546, *p* < 0.001) (Figure 3b).

### 3.2. Topological Analysis of Fungal Community Structures in Mangrove Sediments

**Figure 4:**
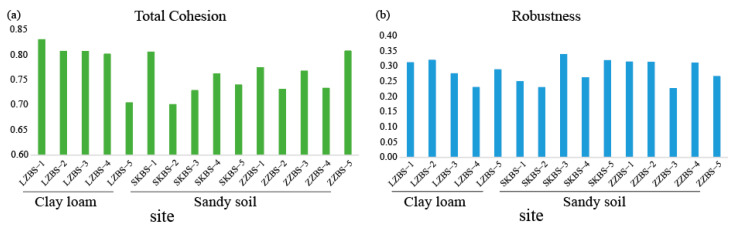
Total cohesion and robustness of fungi in the clay loam and sandy soil in mangrove soils. (**a**): total cohesion; (**b**): robustness.

**Figure 5:**
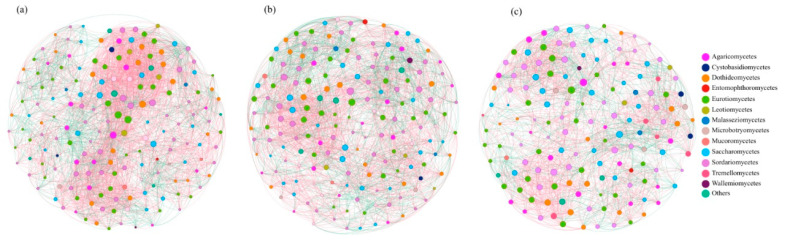
Co-occurrence network of the three groups. (**a**) LZ: Leizhou mangrove site, clay loam; (**b**) SK: Shankou mangrove site, sandy soil; (**c**) ZZ: Zhenzhuwan mangrove site, sandy soil. The color of nodes indicates species from the same module in each network. The line color indicates positive (pink) and negative (green) correlation coefficients.

The degree of community complexity was quantified using the cohesion index [18]. The total cohesion index of the clay loam (0.790) was greater than that of the sandy soil (0.755) (Figure 4a), indicating that the complexity of fungal communities in mangrove clay loam was higher than that in the sandy soil. Moreover, the concentration of community stability was quantified using robustness. The robustness of the clay loam (0.286) was higher than that of the sandy soil (0.284) (Figure 4b), indicating that the stability of fungal communities in mangrove clay loam was greater than that in sandy soil. The clay loam LZ network exhibited the greatest average degree (35.286) and density (0.195), followed by the sandy soil SK network (average degree = 31.05; density = 0.172). These results revealed that OTUs in the clay loam LZ had a relatively greater number of relationships with the other OTUs in the network. While all networks showed the same modules (Modules = 1), the sandy soil ZZ had a greater modularity index (0.575) than LZ (0.518) and SK (0.51) (Table S1). These results indicated that the sandy soil ZZ had stronger anti-interference ability than the clay loam LZ and sandy soil SK (Figure 5).

### 3.3. Environmental Drivers of Fungal Diversity, Complexity, and Stability in Mangrove Sediments

In clay loam samples, Spearman’s correlation analysis revealed that NO_2_^−^-N was the most significant factors influencing the Shannon index (*p* < 0.001) (Table S2). TC was the predominant factor in the clay loam samples (*p* < 0.001) (Table S2). In contrast, C:N, salinity, and PO_4_^3−^ were the most influential drivers in the sandy soil samples (*p* < 0.001) (Table S2).

In the clay loam samples, significant correlations were observed between the variables and the Mantel test, with temperature, C:P, and ORP showing the strongest correlation (*p* < 0.001) (Table S3). In contrast, in the sandy soil samples, pH, TS, and SO_4_^2−^ were the most influential drivers, also displaying significant correlations (*p* < 0.001) (Table S3).

According to Spearman’s rank correlation analysis and the heatmap, in the clay loam samples, NH_4_^+^-N had the strongest impact on complexity, followed by salinity. Total nitrogen was the primary environmental factor affecting stability (Table S4). In contrast, in the sandy soil samples, no environmental factors were significantly correlated (Table S4). However, compared to other environmental drivers, TOC and TP exhibited stronger correlations with complexity and stability, respectively.

**Figure 6:**
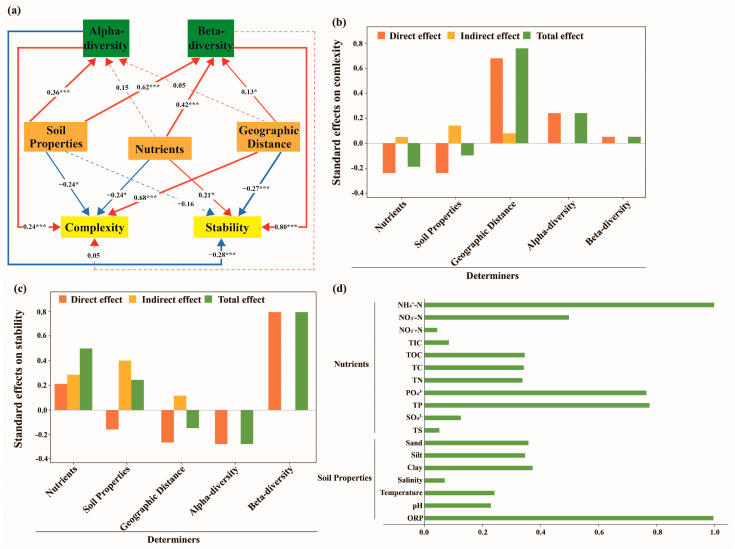
Path analysis representing the relationship between complexity, stability, soil properties, nutrients, alpha diversity, and beta diversity in the clay loam samples. (**a**) Solid and dashed arrows represent significance and insignificance at *p* < 0.05, respectively. Red and blue arrows indicate positive and negative effects, respectively. Significance levels, *: *p* < 0.05; ***: *p* < 0.001, are indicated using different widths of the solid line arrows; the numbers are path coefficients. (**b**) The direct, indirect, and total effects of different factors on the complexity. (**c**) The direct, indirect, and total effects of different factors on the stability. (**d**) The individual factor’s contribution to the module. ORP, pH, temperature, salinity, and soil texture (clay, silt, and sand) are summarized as soil properties. NH_4_^+^-N, NO_2_^−^-N, NO_3_^−^-N, TIC, TOC, TC, TN, PO_4_^3−^, TP, SO_4_^2−^, and TS are summarized as nutrients.

**Figure 7:**
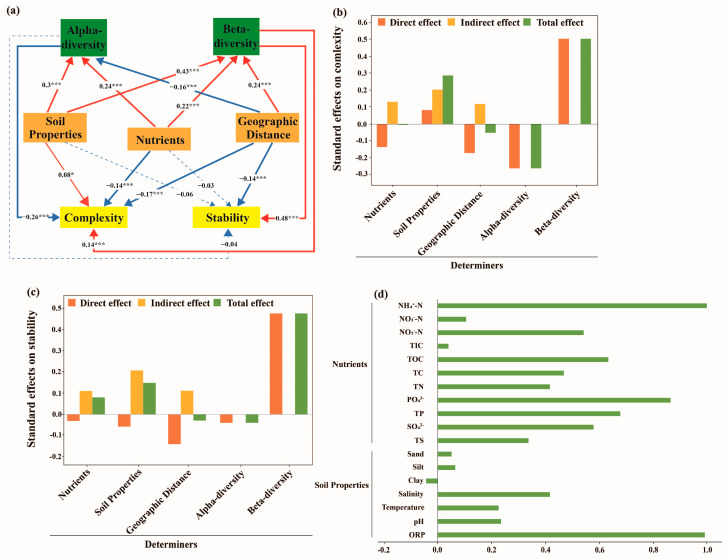
Path analysis representing the relationship between complexity, stability, soil properties, nutrients, alpha diversity, and beta diversity in the sandy soil samples. (**a**) Solid and dashed arrows represent significance and insignificance at *p* < 0.05, respectively. Red and blue arrows indicate positive and negative effects, respectively. Significance levels, *: *p* < 0.05; ***: *p* < 0.001, are indicated using different widths of the solid line arrows; the numbers are path coefficients. (**b**) The direct, indirect, and total effects of different factors on the complexity. (**c**) The direct, indirect, and total effects of different factors on the stability. (**d**) The individual factor’s contribution to the module. ORP, pH, temperature, salinity, and soil texture (clay, silt, and sand) are summarized as soil properties. NH_4_^+^-N, NO_2_^−^-N, NO_3_^−^-N, TIC, TOC, TC, TN, PO_4_^3−^, TP, SO_4_^2−^, and TS are summarized as nutrients.

PLS-PM was constructed to analyze the relationships among complexity, stability, and diversity, as well as soil properties, nutrients, and geographic distance, in different soil types. In clay loam samples, alpha diversity (path coefficients = −0.28, *p* < 0.001) and beta diversity (path coefficients = 0.08, *p* < 0.001) jointly affected the stability of the fungal community structure, with alpha diversity showing a direct and total negative effect, while beta diversity exhibited a direct and total positive effect (Figure 6a,c). Additionally, geographic distance (path coefficients = −0.27, *p* < 0.001) had a greater negative direct effect on the stability, whereas nutrients (path coefficients = 0.21, *p* < 0.05) had a positive direct effect on the stability (Figure 6a,c). For the complexity of fungal community structures in clay loam, alpha diversity (path coefficients = 0.24, *p* < 0.001) showed a direct and total positive effect, whereas beta diversity (path coefficients = 0.05, *p* > 0.05) showed no effect (Figure 6a,b). Moreover, geographic distance (path coefficients = 0.68, *p* < 0.001) had a stronger positive direct effect on the complexity, whereas nutrients (path coefficients = −0.24, *p* < 0.05) and soil properties (path coefficients = −0.24, *p* < 0.05) showed negative effects on the complexity (Figure 6a,b). In the sandy soil samples, alpha diversity (path coefficients = −0.26, *p* < 0.001) and beta diversity (path coefficients = 0.14, *p* < 0.001) were also significantly related to complexity, with alpha diversity showing a direct and total negative effect, whereas beta diversity showed a direct and total positive effect. Nutrients (path coefficients = −0.14, *p* < 0.001) and geographic distance (path coefficients = −0.17, *p* < 0.001) were significantly negatively correlated with complexity, whereas soil properties (path coefficient = 0.08, *p* < 0.05) showed a positive correlation (Figure 7a,b). For stability in sandy soil, beta diversity (path coefficients = 0.48, *p* < 0.001) exhibited a significantly positive direct and total effect, whereas alpha diversity (path coefficient = −0.04, *p* > 0.05) had no effect on stability. Additionally, only geographic distance (path coefficients = −0.14, *p* < 0.001) exerted a stronger negative effect (Figure 7a,c). From the above results, we concluded that alpha diversity emerged as the most influential factor in the complexity and stability of the fungal community structure in the clay loam samples compared to beta diversity, whereas nutrients and geographic distance were identified as the most vital factors for complexity and stability (Figure 6a–d). In contrast, in the sandy soil samples, beta diversity emerged as the most influential factor, affecting both complexity and stability (Figure 7a–d).

## 4. Discussion

### 4.1. Soil Types and Nutrient Availability Influencing Fungal Community Composition and Diversity in Mangrove Sediments

Nutrient availability and soil types significantly affected the composition of fungal communities in mangrove sediments. In the present study, the clay loam and sandy soil samples exhibited different dominant fungal classes. *Sordariomycetes* and *Eurotiomycetes* were commonly found in mangrove sediments, with a high relative abundance in the sandy soil. *Sordariomycetes* and *Eurotiomycetes* are prevalent in coastal ecosystems because of their adaptability to environments with abundant organic material and stress factors such as salt and water temperature [20]. These fungal classes, particularly in mangroves and coastal wetlands, play key roles in the decomposition of organic materials such as plant residues, wood, and cellulose which are prevalent in these ecosystems [21,22]. Their ability to degrade complex organic compounds, such as lignin and cellulose, is especially important in areas rich in organic matter, such as mangrove sediments [23,24]. It is crucial to maintain soil fertility and ecosystem health [25,26]. Sandy soils typically have high aeration and low water retention capacity, which allow certain fungal groups to thrive and grow better in humid, organic-rich environments [27].

In our study, the sandy soil samples exhibited higher alpha diversity, whereas the clay loam samples showed the lowest alpha diversity (Figure 3a). This suggested more ecological niches and greater environmental adaptability in the sandy soil. This may be attributed to the favorable ecological conditions, environmental factors, and soil properties in that area. For example, K Palit et al. [28] and J Lai et al. [29] found that environmental factors like salinity, pH levels, and organic matter content can affect microbial diversity, which aligns with our observations. In contrast, the low diversity in the clay loam samples may reflect the extreme environmental conditions in this region, such as high salinity or low organic matter content, which may constrain the ability of certain fungal communities to thrive, leading to a more homogeneous fungal community in clay loam soils [30]. The results of PCoA and ANOSIM analyses further confirmed that fungal community structures in mangrove sediments exhibited significant differences (Figure 3b,c). These differences can be attributed to the unique properties of mangrove ecosystems and sediments, including variable salinity, organic matter content, and tidal dynamics, which collectively influence fungal community diversity in mangrove soils. These findings showed that fungal communities in mangrove ecosystems are highly dynamic and responsive to soil types and environmental conditions, including hydrodynamic factors, salinity conditions, and spatial distribution [31,32]. Our results suggest that fungal communities display distinct characteristics and variations in response to environmental changes in clay loam and sandy mangrove soils.

### 4.2. Higher Complexity and Stability of Fungal Communities in Clay Loam Compared to Sandy Soil in Mangrove Ecosystems

Cohesion is regarded as an index that reflects the complexity of the fungal community structure, indicating the degree of interaction between the fungal community structures. The cohesion index of clay loam (total cohesion = 0.790) was higher than that of sandy soil (total cohesion = 0.755), suggesting that the complexity of the fungal community structure in clay loam is greater than that in sandy soil. Robustness is also considered an indicator of the stability of the fungal community structure. Our results indicate that the robustness index for clay loam (robustness = 0.286) was greater than that for sandy soil (robustness = 0.284), implying that the fungal community structure in clay loam was more stable than that in sandy soil. These results suggest that the fungal communities in clay loam mangrove soils exhibit more species interactions and a greater ability to adapt to environmental changes [33]. However, K Zhang et al. [34] found that the biodiversity and interactions within microbial communities are crucial determinants of their resistance to and resilience following disturbances. In our research, the sandy soil samples exhibited higher alpha diversity compared to the clay loam. Clay loam has a stronger water retention capacity and lower erosivity, which helps reduce environmental disturbance and provides a more stable habitat under dynamic environmental conditions [35]. Due to the poor nutrient retention capacity of sandy soil [36], fungi may quickly adapt to rapidly changing nutrient environments, which may lead to an increase in the number of certain fungal species, thereby increasing the alpha diversity of the community. In comparison with the sandy soil samples (nodes = 181 and 157; edges = 2810 and 2119), nodes and edges were higher in the clay loam samples (nodes = 182; edges = 3211), which may imply that the soil type, to some extent, determined the ecological structure of mangrove fungal communities. In addition, fungal relationships tend to be more positive and cooperative in clay loams [37]. Therefore, understanding the complexity and stability of fungal communities is crucial for recovering from disturbances and preventing the degradation of mangrove forests.

### 4.3. Main Driving Factors Influencing Complexity and Stability in Clay Loam and Sandy Soil in Mangrove Ecosystems

Our findings revealed a complex interplay between driving factors and fungal community dynamics across soil types in mangrove ecosystems. C Wang et al. [38] suggested that in various interactions, competition for resources is the main factor determining the adaptation and niche differentiation of soil fungal communities. The observed negative correlation between carbon or nitrogen availability (TC, NO_2_^−^-N, and TOC) and fungal diversity (Shannon index) in the clay loam samples aligns with competition for soil resources in ecology. Our study also found that pH was significantly positively correlated with the Shannon index (Table S2), revealing that fungal community diversity was higher in soils with higher pH. Mantel test results demonstrated that temperature, the C:P ratio, and the C:N ratio were crucial environmental driving factors of fungal community composition in mangrove sediments (Table S3). Consistently, H Craig et al. [39] documented that nitrogen addition can alter the composition and diversity of microbial communities in mangrove soils. Z-F Zhang et al. [10] found that carbon cycling supports a variety of adapted microorganisms in terms of abundance and diversity. This may be because higher concentrations of carbon and nitrogen compounds increase resource competition. Our previous results revealed that there is more competition than cooperation among fungi in mangrove soils. J Rousk et al. [40] found that fungi in soil proliferate in acidic environments. However, we observed that in soils with higher pH values, fungal community diversity was higher. This may be due to the stimulation of certain enzymes by neutral (pH = 7) and alkaline (pH = 9) conditions [41], which promoted fungal growth and metabolism. This result may explain why *Eurotiomycetes* and *Sordariomycetes* are dominant in mangrove soils.

In contrast, the Shannon index was significantly positively correlated with salinity and the C:N ratio in the sandy soil samples, demonstrating that higher salinity and C:N ratios might promote fungal diversity (Table S2). In addition, our research revealed that PO_4_^3−^ was significantly negatively correlated with the Shannon index, indicating that the higher the phosphate concentration, the lower the fungal community diversity in mangrove sediments (Table S2). The Mantel test showed that SO_4_^2−^, TC, and TS were important environmental factors driving fungal community composition (Table S3). Similarly, microorganisms play a crucial role in sulfur transformation in mangrove ecosystems [42]. Mangrove plants may absorb sulfate through their root systems [43], influencing the availability of sulfur in the soil, which in turn affects the distribution of fungi. Finally, it alters the composition of the fungal communities. Sulfur may synergize with carbon and nitrogen cycling to shape fungal distribution in mangrove ecosystems. The content of sulfur, carbon, and nitrogen may serve as the comprehensive indicators of the health of mangrove ecosystems.

Soil types emerge as a critical mediator for regulating the complexity and stability of fungal communities in response to environmental drivers. High water retention of clay loam facilitated nitrogen accumulation, especially NH_4_^+^-N and total nitrogen (Table S4), driving intricate fungal networks through competitions for resources [27]. Similar to our findings, H Yang et al. [44] found that nitrogen addition could disrupt the complexity and stability of fungal networks. In contrast, no clear environmental factors influenced the network in sandy soil samples (Table S4). These findings may have been caused by high permeability, leading to rapid nutrient loss and lower nitrogen content. This may be because fungi in mangrove sediments compete for limited resources and exhibit less cooperative behavior. These findings highlight the complex interactions between soil types and fungal network dynamics in mangrove sediments, which are crucial for understanding the ecological functions of mangrove ecosystems, and have significant implications for the restoration, planting, and conservation of mangroves.

PLS–PM analysis revealed that while alpha diversity directly enhanced the complexity of fungal complexity in the clay loam, it weakened the stability of the fungal communities. Fungi can extend their spatial niches to acquire carbon and nutrient resources through the formation of hyphae and mycelia [38], which leads to niche overlap, reduces the efficiency of effective resource allocation, and intensifies competition among species, resulting in an unstable community structure [45]. Beta diversity had a stronger correlation with complexity and stability than alpha diversity in the sandy soil. The impact of beta diversity may enhance community stability by increasing heterogeneity between fungal communities. In conclusion, this study emphasized the main factors driving the complexity and stability of fungal communities, thereby providing theoretical guidance for the biodiversity and protection of mangrove ecosystems.

## 5. Conclusions

In this study, we investigated the fungal community composition and diversity in clay loam and sandy soils in mangrove ecosystems. The alpha and beta diversities exhibited significant divergence between clay loam and sandy mangrove soil samples. Additionally, fungal communities in the clay loam of mangrove soils were more complex and stable than those in sandy soils. NH_4_^+^-N and TN were the main environmental drivers that affected the complexity and stability in the clay loam, respectively. TOC and TP had the greatest correlation (*p* > 0.05) with complexity and stability, respectively, in the sandy soil. PLS-PM analysis demonstrated that alpha diversity limited the complexity and stability of the fungal community and was regulated by soil properties in clay loam. However, the principal limitation on fungal community complexity and stability in the sandy soil was beta diversity. Overall, our research promotes the understanding of how soil types and nutrient dynamics influence fungal community complexity and stability in mangrove ecosystems.

## Data Availability

The original sequence data were deposited in GenBank under BioProject Accession PRJNA771484.

## Supplementary Materials

The following supporting information can be downloaded at https://www.mdpi.com/article/10.3390/jof11040262/s1, Table S1. Topological parameters of the fungal co-occurrence network for the three groups; Table S2. Spearman’s correlation test between the alpha diversity index and environmental factors; Table S3. The Mantel test for correlations between the beta diversity index and environmental factors; Table S4: Correlation among complexity, stability, and environmental factors.

## Abbreviations

The following abbreviations are used in this manuscript:

DO	Dissolved oxygen
TC	Total carbon
TOC	Total organic carbon
TP	Total phosphorus
TS	Total sulfur
TN	Total nitrogen
TIC	Total inorganic carbon
ORP	Oxidation-reduction potential
PCoA	Principal coordinate analysis
ANOSIM	Analysis of Similarity
