# Supplementary material for: Fungal Community Complexity and Stability in Clay Loam and Sandy Soils in Mangrove Ecosystems
Source: J Fungi (Basel). 2025 Mar 28;11(4):262. doi: 10.3390/jof11040262 (PMC12028037; doi:10.3390/jof11040262)
Supplement: Supplementary file 1 [file jof-11-00262-s001.zip › jof-3479874-supplementary.pdf]

Table S1. Topological parameters of the fungal co-occurrence network for the three groups.

Metri	LZ (Clay Loam)	SK (Sandy Soil)	ZZ (Sandy Soil)
Nodes	182	181	157
Edges	3211	2810	2119
Average Degree	35.286	31.05	26.994
Diameter	4	3	4
Density	0.195	0.172	0.173
Clustering Coefficient	0.676	0.602	0.648
Modularity	0.518	0.51	0.575
Modules	1	1	1
Positive Edges	2355	1770	1410
Negative Edges	856	1040	709

Table S2. Spearman's correlation test between alpha diversity index and environmental factors. ORP: Oxidation-reduction potential; pH: pH value; Temp: Temperature; Sal: Salinity; TS: Total sulfur; TP: Total phosphorus; TN: Total nitrogen; TC: Total carbon; TOC: Total organic carbon; TIC: Total inorganic carbon; C:N: Carbon-to-nitrogen ratio; NO_2^--N: Nitrite-nitrogen; NO_3^--N: Nitrate-nitrogen; NH_4^+-N: Ammonium-nitrogen; MNI: Minimum number of individuals; RD: Resource Diversity; C:P: Carbon-to-phosphorus ratio; N:P: Nitrogen-to-phosphorus ratio; C:N:P: Carbon-to-nitrogen-to-phosphorus ratio; *: $p < 0.05$; **: $p < 0.01$; ***: $p < 0.001$.

	Clay Loam	Sandy Soil
	Shannon	Shannon
ORP	−0.35	−0.29 *
pH	0.59 **	−0.24
Temperature	0.29	0.05
Salinity	−0.2	0.6 ***
TS	−0.4 *	0.03
SO_4^{2-}	−0.49 *	0.17
TP	−0.1	−0.09
PO_4^{3-}	0.1	−0.58 ***
TN	−0.15	−0.27
TC	−0.65 ***	−0.19
TOC	−0.59 **	−0.25
TIC	−0.31	0.31 *
C:N	−0.49 *	0.71 ***
NO_2^--N	−0.69 ***	−0.31 *
NO_3^--N	0.49 *	−0.04
NH_4^+-N	−0.39	−0.28 *
MNI	−0.39	−0.3 *
RD	−0.1	0.24
C:P	0.1	0.01
N:P	0.29	−0.27
C:N:P	0.1	0.19

Table S3. Mantel test for correlations between beta diversity index and environmental factors. ORP: Oxidation-reduction potential; pH: pH value; Temp: Temperature; Sal: Salinity; TS: Total sulfur; TP: Total phosphorus; TN: Total nitrogen; TC: Total carbon; TOC: Total organic carbon; TIC: Total inorganic carbon; C:N: Carbon-to-nitrogen ratio; NO_2^--N: Nitrite-nitrogen; NO_3^--N: Nitrate-nitrogen; NH_4^+-N: Ammonium-nitrogen; MNI: Minimum number of individuals; RD: Resource Diversity; C:P: Carbon-to-phosphorus ratio; N:P: Nitrogen-to-phosphorus ratio; C:N:P: Carbon-to-nitrogen-to-phosphorus ratio; ***: $p < 0.001$.

	Clay Loam	Sandy Soil
	Mantel	Mantel
ORP	0.6 ***	0.46 ***
pH	0.58 ***	0.49 ***
Temperature	0.61 ***	0.48 ***
Salinity	0.52 ***	0.44 ***
TS	0.43 ***	0.49 ***
SO_4^{2-}	0.55 ***	0.49 ***
TP	0.53 ***	0.3 ***
PO_4^{3-}	0.55 ***	0.25 ***
TN	0.57 ***	0.47 ***
TC	0.52 ***	0.5 ***
TOC	0.48 ***	0.48 ***
TIC	0.47 ***	0.29 ***
C:N	0.59 ***	0.48 ***
NO_2^--N	0.49 ***	0.33 ***
NO_3^--N	0.32 ***	0.42 ***
NH_4^+-N	0.51 ***	0.28 ***
MNI	0.58 ***	0.28 ***
RD	0.52 ***	0.45 ***
C:P	0.61 ***	0.13 ***
N:P	0.5 ***	0.13 ***
C:N:P	0.55 ***	0.13 ***

Table S4. Correlation among complexity, stability, and environmental factors. ORP: Oxidation-reduction potential; pH: pH value; Temp: Temperature; Sal: Salinity; TS: Total sulfur; TP: Total phosphorus; TN: Total nitrogen; TC: Total carbon; TOC: Total organic carbon; TIC: Total inorganic carbon; C:N: Carbon-to-nitrogen ratio; NO_2^--N: Nitrite-nitrogen; NO_3^--N: Nitrate-nitrogen; NH_4^+-N: Ammonium-nitrogen; RD: Resource Diversity. *: $p < 0.05$; **: $p < 0.01$; ***: $p < 0.001$.

	Clay Loam		Sandy Soil	
	**Complexity**	**Stability**	**Complexity**	**Stability**
ORP	0.46	0.56	−0.27	0.21
pH	−0.5	−0.4	−0.13	0.12
Temperature	0.4	−0.3	−0.22	−0.16
Salinity	0.9 *	0.8	0.02	−0.06
TS	−0.36	−0.1	−0.34	−0.08
SO_4^{2-}	0.1	0.3	0.25	0.09
TP	0.8	0.5	0.17	−0.39
PO_4^{3-}	−0.8	−0.5	0.43	0.22
TN	0.56	0.97 **	0.28	0.22
TC	0.41	0.67	0.14	0.12
TOC	0.5	0.4	0.55	0.18
TIC	−0.26	0.32	−0.22	−0.39
C:N	0.1	0.3	−0.48	−0.2
NO_2^--N	0.1	−0.5	−0.37	0.36
NO_3^--N	0.3	−0.1	0.49	−0.2
NH_4^+-N	1 ***	0.6	0.33	0.12
